# Living with Fibrodysplasia Ossificans Progressiva: Radiological Images of a Patient with Extensive Heterotopic Ossification

**DOI:** 10.3390/diagnostics13101711

**Published:** 2023-05-12

**Authors:** Mohammed Mostafa Kotb, Usama Farghaly Omar, Arun-Kumar Kaliya-Perumal

**Affiliations:** 1 Department of Orthopedics and Traumatology, Reconstructive Microsurgery Unit, Assiut University School of Medicine, Assiut 71526, Egypt; 2 Department of Orthopaedic Surgery, Hand and Reconstructive Microsurgery Service, Khoo Teck Puat Hospital, Singapore 768828, Singapore; 3 Lee Kong Chian School of Medicine, Nanyang Technological University, Singapore 636921, Singapore

**Keywords:** fibrodysplasia ossificans progressiva, heterotopic ossification, ectopic bone, stone man syndrome, myositis ossificans

## Abstract

Fibrodysplasia ossificans progressiva (FOP) is an exceptionally rare genetic disorder characterized by the progressive formation of heterotopic bone in soft tissues. Here, we present the radiological findings of an 18-year-old female diagnosed with FOP who had severe spinal and right-upper-limb abnormalities. Her SF-36 scores suggested significant impairment in physical function, affecting work and other regular daily activities. Radiographic evaluation with X-rays and CT scans revealed scoliosis and total fusion of almost all levels of the spine, with only a few disc spaces spared. A large mass of heterotopic bone was observed, corresponding to the location of the paraspinal muscles in the lumbar region, branching upwards and fusing with the scapulae on both sides. On the right side, this exuberant heterotopic bone mass fused with the humerus, resulting in a fixed right shoulder, while the rest of the upper and lower limbs are spared and have a range of motion. Our report highlights the extensive ossification that can manifest in patients with FOP, resulting in restricted mobility and a poor quality of life. While there is no definite treatment that can reverse the effects of the disease, preventing injuries and minimizing iatrogenic harm is of critical importance in this patient as inflammation is known to play a key role in triggering heterotopic bone. Meanwhile, ongoing research into therapeutic strategies holds the key to unlocking a potential cure for FOP in the future.

**Figure 1:**
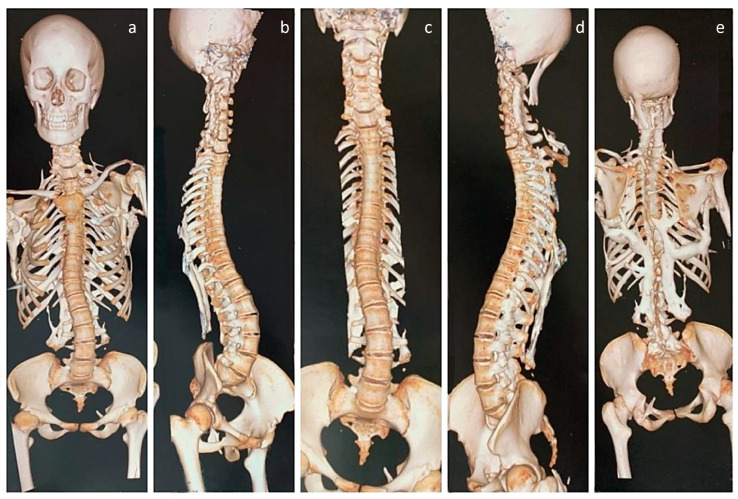
Three-dimensional CT images of the upper body, pelvis, and hip joints of an 18-year-old female with a clinicoradiological diagnosis of fibrodysplasia ossificans progressiva (FOP), an exceptionally rare genetic disease affecting 1 in 2 million people that is caused by a gain-of-function mutation in the gene that encodes activin A receptor type I (ACVR1) [1,2]. The disease is characterized by progressive heterotopic ossifications in various extraskeletal sites [3]. In the patient reported here, the disease initially presented as neck stiffness, followed by multiple episodes of back stiffness, the appearance of notable hard lumps, and finally, restricted movement in the right shoulder joint, with pain preceding stiffness in all affected locations. (**a**–**d**) Thoracolumbar scoliosis and complete ossification of the disc spaces in the thoracic spine can be noted in both the anterior and oblique views, while fusion of the posterior elements, especially at the lumbar region, is evident on the oblique views. (**b**,**d**) The cervical spine appears to be fused, as indicated by fused lateral masses and posterior elements. (**d**,**e**) Heterotopic bone is observed, descending from the occiput, potentially indicating an ossified capitus muscle on the left side. (**e**) On the posterior aspect, a large mass of heterotopic bone is observed, corresponding to the location of the paraspinal muscles, branching upwards and fusing with the scapulae on both sides. On the right side, the heterotopic bone mass is fused with the humerus. (**a**,**e**) In addition, small, heterotopic bony projections are seen, originating from the first rib and pointing upwards. The rest of the upper limb was unaffected, allowing for full active range of motion in both elbows, wrists, and hands. Physical function, as measured by the SF-36 score, showed significant impairment (20/100), which affected her work and daily activities. As a result, her emotional and social well-being were also impacted. There were no obvious soft-tissue flare-ups at the time of presentation; however, she had hip pain that was described as moderate and interfering with daily activities.

**Figure 2:**
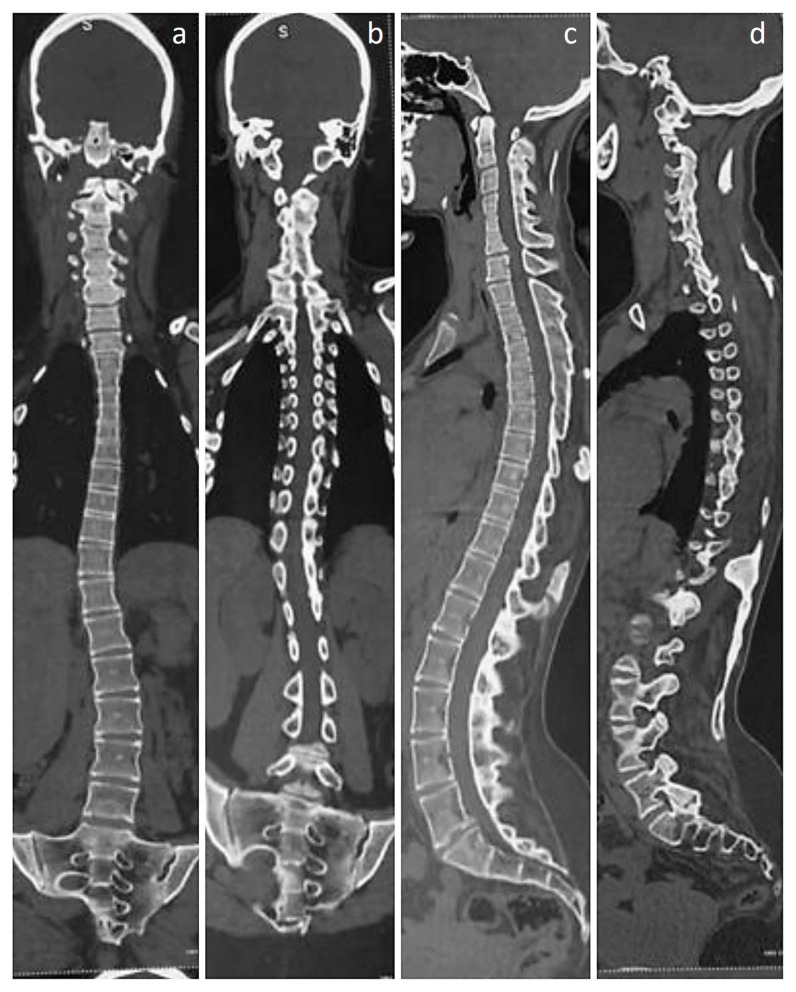
Whole-spine CT scans, both coronal and sagittal sections. (**a**–**d**) Sections clearly show the thoracolumbar scoliosis, along with fused cervical disc spaces and posterior elements, with only the discs above and below C7 appearing to be spared. (**c**) Sagittal section shows the fusion of thoracic disc spaces and the thoracolumbar posterior elements. (**c**,**d**) The heterotopic bone involving the posterior muscles are also seen clearly in the sagittal views.

**Figure 3:**
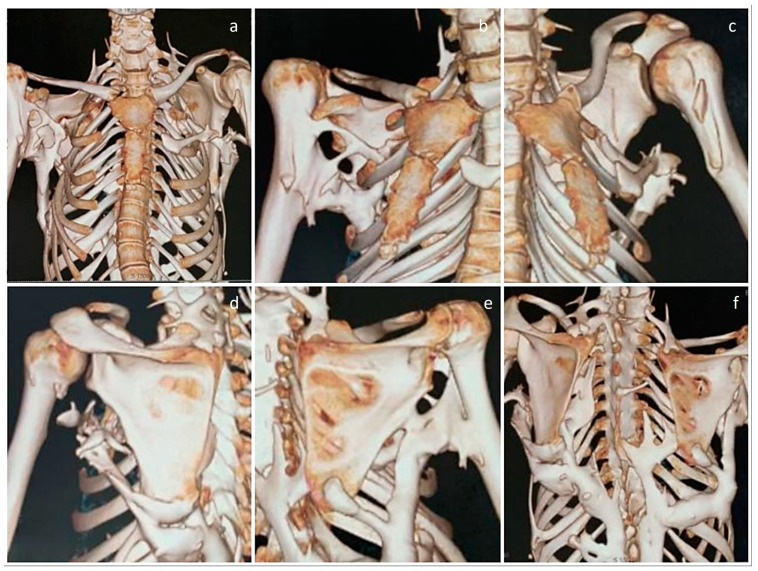
Close-up 3D CT images of the rib cage, scapulae, and shoulder joints. (**a**) While the front of the chest wall appears to be relatively spared, there is an isolated mass of heterotopic bone on the left anterior chest wall adjacent to the sternum. (**b**) Oblique view of the chest wall, showing fusion of the right humerus to the heterotopic bone mass in the posterior aspect. (**c**,**d**) Oblique view, showing that the left shoulder joint and humerus are free of restricting ossifications. (**d**,**e**) Posterior views, showing that both scapulae are fused with the heterotopic bone extending from the lumbar paraspinal region. (**e**) Fusion of the right humerus to the heterotopic bone mass can be noted. (**f**) Posterior view of the chest wall, clearly showing fusion of the posterior elements of the spine.

**Figure 4:**
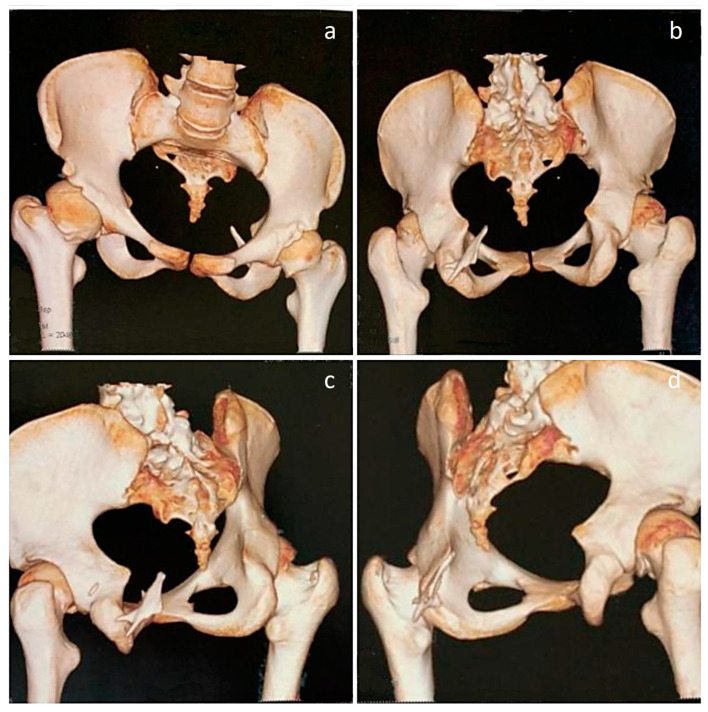
Three-dimensional CT images of the pelvis. (**a**,**b**) Images demonstrate a tilted pelvis, in compensation for the thoracolumbar scoliosis. (**b**–**d**) Posterior and oblique views, showing a heterotopic bony mass arising from the left ischium and projecting upwards. As such, the hip joints are unaffected by movement-restricting heterotopic ossifications; however, asymmetry of femoral heads can be noted, with the right femoral head appearing larger than the left. While hip rotations were restricted to a certain extent, no other heterotopic ossifications were observed in the lower limbs. Despite the significant impairment of mobility caused by widespread ossifications in the spine and the right upper limb, the patient can walk and manage her daily tasks with assistance. Although genetic sequencing was not performed to ascertain the mutation, classical signs were confirmatory of the diagnosis and currently, treatment is focused on mitigating inflammation with the use of nonsteroidal anti-inflammatory drugs and reducing pain [4]. While there is no definite treatment that can reverse the effects of the disease [5], Kaplan et al. have provided a comprehensive overview of the present medical management strategies, which serves as a guide for healthcare professionals treating individuals with FOP [6]. However, it should be noted that there could be progressive development of heterotopic bone at multiple sites, eventually leading to a significant loss of mobility [7,8]. Hence, it is important for patients to work closely with healthcare providers to manage symptoms and maintain the highest possible level of function and quality of life. While ongoing research on novel drugs targeting the FOP process continues, it is essential for clinicians to be aware of the initial diagnostic features of the condition, such as malformed great toes, even prior to the onset of heterotopic ossification [9]. Doing so gives a chance for early genetic consultation and testing. If signs favor a diagnosis of FOP, preventing injuries and minimizing iatrogenic harm is of critical importance, as injury sites are known to be susceptible to the cascade of events that lead to heterotopic ossification [10].

## Data Availability

Not applicable.
